# The Clinicians’ Guide to Large Language Models: A General Perspective With a Focus on Hallucinations

**DOI:** 10.2196/59823

**Published:** 2025-01-28

**Authors:** Dimitri Roustan, François Bastardot

**Affiliations:** 1 Emergency Medicine Department Cliniques Universitaires Saint-Luc Brussels Belgium; 2 Medical Directorate Lausanne University Hospital Lausanne Switzerland

**Keywords:** medical informatics, large language model, clinical informatics, decision-making, computer assisted, decision support techniques, decision support, decision, AI, artificial intelligence, artificial intelligence tool, LLM, electronic data system, hallucinations, false information, technical framework

## Abstract

Large language models (LLMs) are artificial intelligence tools that have the prospect of profoundly changing how we practice all aspects of medicine. Considering the incredible potential of LLMs in medicine and the interest of many health care stakeholders for implementation into routine practice, it is therefore essential that clinicians be aware of the basic risks associated with the use of these models. Namely, a significant risk associated with the use of LLMs is their potential to create hallucinations. Hallucinations (false information) generated by LLMs arise from a multitude of causes, including both factors related to the training dataset as well as their auto-regressive nature. The implications for clinical practice range from the generation of inaccurate diagnostic and therapeutic information to the reinforcement of flawed diagnostic reasoning pathways, as well as a lack of reliability if not used properly. To reduce this risk, we developed a general technical framework for approaching LLMs in general clinical practice, as well as for implementation on a larger institutional scale.

## Introduction to Large Language Models

The development of artificial intelligence (AI) solutions and their recent democratization have allowed the public to access various innovative tools. Notably, several large language models (LLMs) have recently surged in popularity due to significant media attention and by offering free access for registered users (eg, ChatGPT, Gemini, and Meta LLaMA).

An LLM is a type of deep learning model that is pretrained on large text datasets. They are often based on the transformer architecture [1], an innovative form of neural network that uses an encoder-decoder structure to rapidly process large blocks of text, avoiding redundancies that hampered recurrent neural networks in the past. Several popular LLMs have integrated a chatbot interface to allow users to interact directly with the model, generating appropriate, context-aware responses to a user’s input in a conversational manner. This allows the user to engage in dynamic conversations that appear natural, making the technology a powerful tool for various applications across a wide range of fields.

The use of LLMs has become widespread, and medicine is no exception. LLMs have the potential of becoming a disruptive tool in medicine [2] and will certainly have a major impact on clinical practice, medical education, and research. Within this field, LLM performance has already been evaluated to take specialist board exams [3], improve communication with patients [4,5], and write drafts for scientific papers. It can also be an interesting tool for creating a dynamic learning experience [6]. Although LLMs with chatbot interfaces represent an interesting tool for many tasks in clinical medicine and research, users should be aware of one of the most significant shortcomings of these models, called “hallucinations.”

In this viewpoint, we review the underlying causes of hallucinations in LLMs and examine their implications within the field of clinical medicine. We also explore current and future strategies for mitigating these limitations and present a general framework to guide clinicians in critically assessing and integrating LLMs into clinical practice.

## Overview of Hallucinations

In medicine, hallucinations refer to sensory experiences that occur in the absence of corresponding external stimuli. In the field of LLMs, hallucinations refer to the generation of false or fabricated information. This signifies that the LLM will create nonfactual content to answer a user’s question without clarifying whether the answer contains fabricated information. Hallucinations stem from many root causes, which we will delve into below.

First, both the quality and volume of the dataset upon which the LLM has been trained are important variables and can explain the number of hallucinations the LLM produces to some degree [7,8]. How data are collected and how the model is trained can also influence hallucination frequency [8]. Furthermore, the method through which the editor fine-tunes the model can also influence the final output.

Another major cause of hallucinations stems from the very way certain LLMs are programmed. Indeed, most LLMs are auto-regressive; the term “auto-regressive” refers to the model’s ability to predict future elements of a sequence based on its previous outputs. These elements, usually one or multiple words, are termed tokens. An auto-regressive LLM aims to produce an output based on token prediction; this signifies that the model will predict the most probable next token(s) given a specific input token. In practice, it predicts the following word(s) after the sequence of words it has already given. However, it generates each next token by considering the previous one, and not the whole sequence. This means words are generated in a word-after-word fashion, without necessarily using the whole of the previously generated sentence to predict the rest of the sentence [9]. This lays the groundwork for producing hallucinations since factual accuracy is not the end goal. Rather, accuracy is inferred from a high probability of adequate token prediction based on the data in the training dataset. Since the dataset is necessarily flawed or incomplete, hallucinations can arise.

The size of the training dataset can also influence hallucination type and degree. It has been demonstrated on multiple LLMs that the larger the training dataset size, the more likely the model will be capable of recognizing its limitations and acknowledging uncertainty [10]. Furthermore, choices made by the editor will also influence output quality (ie, fine-tuning decisions, output ranking, censorship, etc).

User input is also of great importance in determining the quality of the model output. Indeed, it has been shown that user input through contextualization and inclusion of source material can also modify the number of hallucinations an LLM produces [11].

## Implications for Clinical Medicine

LLMs have many potential benefits in the health care system, for both providers and patients. For simple tasks, it is highly likely that part or all of the process will be carried out with LLM tools in the foreseeable future. Efficiency will likely be improved by reducing redundant and tedious tasks (most likely administrative before clinical) [12-14], and there may even be applications for reducing diagnostic delay for difficult diagnoses [15]. Nonetheless, despite these potential benefits, hallucinations represent a major risk if unaccounted for when using LLMs [16-18]. Below, we will review some situations that have appeared apparent to us when testing LLMs.

In practice, LLMs may erroneously attribute clinical, biological, or radiological characteristics to certain diseases or conditions, depending on the way the clinician inputs data as well as the probabilistic behavior of the model. This flaw, in combination with anchoring and confirmation bias, may unknowingly lead the clinician down an erroneous diagnostic or therapeutic pathway. This can have severe consequences for the patient’s health.

LLMs may also make false claims about diagnostic accuracy for diagnostic procedures. This can lead the clinician to either overestimate or underestimate the diagnostic capacity of a procedure. The consequences could be either depriving a patient of a reliable diagnostic method or, on the contrary, relying on an inadequate diagnostic method to make a statement about the disease process. More specifically in the latter case, the absence of a disease process may be wrongly inferred based on an insensitive exam, and the presence of a disease process may be improperly inferred from a nonspecific exam.

Furthermore, the LLM may suggest inadequate workups and therapeutic procedures. It is important to remember that LLMs are trained on databases that may either not encompass the data necessary to provide adequate guidance (ie, absence of medical guidelines) or contain outdated medical recommendations. Further, given the crucial importance of input data supplied by the user, the omission of a simple characteristic may cause the LLM to produce an inadequate plan of care. Moreover, the LLM may not necessarily prompt the user for additional information regarding important characteristics that could influence the plan of care; most notably, social characteristics and cultural preferences may be inadequately accounted for. Specific diagnostic or therapeutic measures proposed by the LLM may be inadequate or inappropriate, based on important parameters such as pretest probability, as well as patient preferences and prognosis. In addition, given the diversity of health care system models in countries around the world, the inherent bias introduced by the LLM’s dataset can lead to recommending inadequate plans of care for a different health care model than that which the dataset contains information on.

Consistency, and thus reliability, is another issue that can appear while using LLMs to make recommendations for plans of care. Indeed, even with a consistently identical user query, the information contained within the LLMs response may vary considerably when the user renews the query. This variability is an important consideration when the clinician is contemplating the possibility of integrating LLMs into the patient care process. Indeed, it is well accepted in the health care quality community that reducing variability to a certain degree is an essential step in increasing quality [19]; this is even more so true when some parts of the information provided by the LLM are at risk of being inadequate.

In addition, the use of LLMs in clinical practice raises a significant number of ethical concerns, which we only slightly touch upon in this viewpoint. Questions regarding overreliance on LLMs and other AI tools, as well as the legal and ethical ramifications of decision-making based upon AI input, are crucial. In the era of evidence-based medicine, LLM source material and information traceability will be essential in order to reliably inform patient care decisions. The value of the clinician’s experience in nuancing the LLM’s outputs will also remain critical in delivering personalized patient care.

To build upon the legal and ethical concerns related to these LLMs, it is important to keep in mind that many LLMs are developed by private companies and are not open source. Data management, especially related to patient privacy rights, is an essential concern related to information input into the LLM. Implementation of LLM components in electronic medical record programs is being considered and poses the same risks. Notably, the question stands on the use of personal data to for-profit ends (targeted advertising or selling health care data to insurance companies). Local institutional governance and national regulations will be essential in managing and mitigating these potential risks.

Finally, LLM use also carries considerable potential to alter the patient-clinician relationship. Patients may increasingly discuss their symptoms and conditions with LLMs before seeing a medical professional, in a similar way that some patients use search engines before consulting a physician today. This poses the risk of fostering misguided self-diagnoses, with potentially harmful health consequences, especially in settings where access to health care can be financially challenging. Furthermore, patients might develop unrealistic expectations or demand unnecessary clinical resources, based on the information the LLM has provided. As a result, this could affect the dynamics of the patient-clinician relationship. On the other hand, LLMs possess the capacity to tailor medical information to the patient’s level of comprehension, which may help enhance therapeutic education and adherence to medical advice.

## Mitigating Hallucinations in Clinical Practice

Hallucinations can therefore represent a significant source of error if unaccounted for when using LLMs. A proactive and systematic approach is necessary to help interpret LLM output data and avoid succumbing to avoidable pitfalls that could cause harm to patients. This approach is summarized in Textbox 1.

**Important technical considerations before integrating large language models (LLMs) into clinical practice.**

- What dataset was the LLM trained upon?
  What specific considerations does this entail, with regard to bias?
  Is the dataset up to date?
- What organization is behind the LLM and the dataset?
- Is the LLM specifically tailored for medical purposes?
- In testing rounds:
  Is the information given by the LLM consistent with the existing knowledge on the subject?
  Are the recommendations made by the LLM adequate compared to the accepted standard of care?
- How much variability exists within the LLM’s responses? Can it be clinically significant for patient care?
- Enhanced capacity:
  - Does the model possess the capacity to integrate up-to-date information?
  - Does the model possess the capacity to search within reliable sources of information to better respond to the user’s request? If the answer to one of the two above questions is yes, is this feature integrated within the LLM or is it operated by a third-party plug-in?
    - If a third-party plug-in is involved, what strengths and shortcomings does it entail?
  - Does the LLM possess the capacity to assess its answers’ reliability?
  - Is the LLM capable of providing the links to its sources of information?
- Do best practice guidelines exist for the utilization of the LLM?
  - If so, are they specific to use in the health care sector?
  - If the guidelines are general use or specifically focused on another industry sector, what precautions must be applied before extrapolating their use to the health care sector?
- Has the model been tested in a rigorous fashion, and are the results of this evaluation subject to scientific publication?

First and foremost, understanding the model’s origins, version, training database content, and strengths as well as drawbacks are essential prerequisites for an informed use of the LLM. With this information, the user should actively seek out what types of bias the model may contain and understand how it can affect the LLM’s answers [2]. Furthermore, information on the training dataset should be sought out to understand how up-to-date the knowledge within it is, as well as if it is well equipped to answer medical inquiries. In this regard, a topic of emerging importance is the development of LLMs specifically trained for medical purposes. Although theoretically more performant than general LLMs, their relevance for clinical practice has not yet been evaluated.

Second, user input should be carefully crafted to create a high-quality request. The request should contain a detailed description of the clinical context; this requires carrying out a thorough history, clinical exam, and incorporating current as well as historical workup data. Clinical acumen thus remains essential in creating an adequate request. Therefore, although initially time-consuming, a higher-quality request can yield a more relevant answer.

Third, model accuracy and hallucination prevalence should be assessed before being put into practice, through iterative testing and evaluation. During testing rounds, LLM accuracy should be examined using standardized scenarios. Consistency, as well as variability in the answers, should be evaluated by regenerating the LLM’s responses multiple times. Whether through formal, statistical evaluation or through getting a general sense of the model’s characteristics, the clinician can evaluate the LLM’s capabilities and shortcomings in this manner. Furthermore, the scientific adequacy of the LLM’s responses should be assessed with regard to current standards of care and up-to-date guidelines. Even without knowing the database’s knowledge cutoff, this method can help assess how up-to-date the data are, as well as understand how often hallucinations arise with regard to a specific subject.

Another useful tool that can help the clinician assess the reliability of the LLM’s responses is plug-ins. These are usually third-party apps that can be programmed to serve a wide range of functions, including but not limited to, searching the internet, retrieving information from scientific databases, and substantiating responses with links to the sources of the presented information. LLMs may also possess certain of these capacities directly within the scope of their own functions. Although plug-ins may significantly enhance the reliability of the LLM’s responses, by providing the ability for up-to-date referencing, they are not a guarantee that the response will be free from hallucinations. Therefore, plug-ins should be evaluated with the same amount of scrutiny as the LLM itself.

Finally, combining text, image, and video data in LLM training databases can lead to more accurate responses and may decrease the likelihood of hallucinations [20]. However, it is also important to remember that the multimodal model’s performances still rely on the quality of their training dataset [21].

A crucial aspect in the deployment and judicious implementation of LLMs in a clinical setting lies in the establishment of a proactive error reporting program. In conjunction with the aforementioned recommendations, the implementation of such a program facilitates the identification and reporting of near-miss incidents. At the individual level, this allows the user to develop a personal appreciation of the model’s shortcomings, as well as the topics subject to hallucinations. On an institutional level, it can help develop best practice guidelines by identifying frequent hallucination presentations and more general errors. If LLM solutions are delivered as an on-premises solution, it is conceivable that error reporting will help fine-tune local models.

## Prospects

Research in AI will largely contribute to reducing hallucinations, be it through fine-tuning of the underlying model, prompt engineering techniques, development of specific medical LLMs, or other innovative approaches.

Given the increasing awareness of hallucinations, and understanding the risk they potentially pose to patient safety, ingenious mitigation strategies have recently developed. Measurement of semantic entropy [22], algorithmic approaches to address root causes of hallucinations [23], and more classical methods such as Retrieval-Augmented Generation [24] are some of the many techniques that have been proposed to identify and reduce hallucinations. Ideally, an automated combination of different strategies may help both accurately identify and reduce the occurrence of hallucinations. This would help ensure LLM response accuracy and reliability.

To remain up-to-date with these rapid and substantial developments in technology, many approaches will be required to ensure clinicians stay current and use these tools to the best of their capacity. Specific health care–related research will be required to evaluate the full extent of individual LLM capacities and performance. Furthermore, in the same way clinicians require continuing education in emerging and evolving health care topics, frequent training will be essential to use LLM-related tools adequately. Particular attention should be directed towards recognizing and mitigating the numerous pitfalls associated with their use. To this end, we have identified significant practical limitations of LLMs that may limit their rapid uptake into daily clinical practice (Textbox 2).

**Factors currently limiting the widespread implementation of large language models (LLMs) in clinical settings.**

**Model related**

- Risk of bias related to:
  The quality and quantity of the data used to train the model
  How the model was trained and especially fine-tuned
  How the LLM reacts to different methods of prompting
- Difficulty of complex models to explicit the reasoning behind their responses
  Hallucination risk, without the capacity to inform the user on the final output’s trustworthiness: information related to the source of the information not necessarily provided
  No assessment of source reliability

**Human related**

- Risk of misuse related to:
  Idealization of LLM capacities, and considering them to be completely foolproof, may lead to expert bias
  Biased user input and uncritical approval of LLM output, aligning with the user’s anticipated answer, can result in confirmation bias
  Increased dependency on automated aids without critical thinking and reassessment, can lead to automation bias
- Absence of a strong legal framework defining the scope and regulatory environment of LLMs, as of date
- Lack of institutional governance defining the following:
  Methods of informing patients and obtaining their consent for use of LLMs in their health care pathway
  Integration of LLM use within a legal framework
  Accountability in case of an error resulting from the use of the LLM
  Methods of initial testing, implementation, and continuous improvement of the selected LLM
  General operating conditions, namely:
    - Defining specific tasks for which the LLM should be used
    - Ensuring a human has the final word in the decision-making process, even though it is assisted by the LLM
  Training procedures and certifications required for health care professionals to use the tool
  Implementation format (on-premises vs outsourced)
  Quality control processes
  Traceability of feedback and changes in LLM implementation
  Ethical framework

**Economically related**

- Cost of the initial investment
- Cost related to maintenance in an “on-premises” format, including:
  Employee wages
  Infrastructure costs
  Energy costs
- Concerns regarding sustainability, given the high energy consumption of servers used to power and train current LLM models

As previously mentioned, future iterations of LLMs could also be specifically trained on medical datasets and fine-tuned by expert clinician input. This could be an effective method of reducing hallucinations and would allow the tailoring of LLMs to specific fields of medicine.

## Conclusions

Due to its vast potential, LLM integration into routine clinical practice is no longer a question of if, but when. As technology advances, the integration of LLMs with other AI tools possessing multimodal analysis capabilities (text, audio, and image) will follow suit. These advances offer significant opportunities in terms of patient care. However, robust legal frameworks will be necessary to guide their use on a national scale, and institutional governance is key to their implementation for everyday use. Indeed, an informed approach to using these tools, as well as significant efforts in terms of capacity-building, are primordial to avoid falling victim to their well-identified shortcomings.

## Abbreviations

AI: artificial intelligence
LLM: large language model
